# Overconfidence, Incentives and Digit Ratio

**DOI:** 10.1038/srep23294

**Published:** 2016-04-04

**Authors:** Levent Neyse, Steven Bosworth, Patrick Ring, Ulrich Schmidt

**Affiliations:** 1 Kiel Institute for the World Economy, Germany; 2 Department of Psychology, Kiel University, Germany; 3 Department of Economics, Kiel University, Germany; 4 Department of Economics and Econometrics, University of Johannesburg, South Africa

## Abstract

This paper contributes to a better understanding of the biological underpinnings of overconfidence by analyzing performance predictions in the Cognitive Reflection Test with and without monetary incentives. In line with the existing literature we find that the participants are too optimistic about their performance on average; incentives lead to higher performance; and males score higher than females on this particular task. The novelty of this paper is an analysis of the relation between participants’ performance prediction accuracy and their second to fourth digit ratio. It has been reported that the digit ratio is a negatively correlated bio-marker of prenatal testosterone exposure. In the un-incentivized treatment, we find that males with low digit ratios, on average, are significantly more overconfident about their performance. In the incentivized treatment, however, we observe that males with low digit ratios, on average, are less overconfident about their performance. These effects are not observed in females. We discuss how these findings fit into the literature on testosterone and decision making and how they might help to explain seemingly opposing evidence.

Social comparison theory suggests that individuals make predictions in both social and individual contexts every day^1^. For example, the decision to participate in a costly competition is driven by predictions about one’s own abilities relative to the competitors’^2^,^3^. While under certain circumstances it appears beneficial to base such a decision on accurate judgments, in some situations it may be advantageous to be overconfident^4^. Different types of overconfidence have been observed and discussed in the literature^5^.

Among other things, overconfidence can have a positive impact on one’s own motivation and a detrimental impact on competitors’ motivation^6^,^7^,^8^. If this is the case, overconfidence increases the probability of success and therefore could be seen as an adaptive survival strategy^9^. Empirically, overconfidence has a positive impact on success in academia^10^ and war^11^. Additionally, it seems to promote human well-being by giving oneself a positive view of the future^12^,^13^. It does not, however, increase the chances of success in every situation, because overconfident beliefs are by definition inaccurate^14^. Therefore, they can be exploited by rational agents^15^. For instance, overconfident investors trade too much and thereby reduce their earnings^16^. Overly optimistic beliefs about the future potentially explain the high rates of business failure^17^. These findings suggest that the adaptiveness of overconfidence is highly sensitive to the strategic context in which it is deployed.

Despite this trade-off, overconfident judgments are a manifested trait of human decision making^18^. This trait is not balanced across genders however. While both males and females can be overconfident on average, males’ beliefs tend to be even more biased^19^. Gender differences in overconfidence, for example, can have substantial impacts on willingness to compete: Men are more likely to select into tournament compensation schemes than women, and male overconfidence explains a large share of this gap^2^.

Based on the gender difference found in confidence levels, the sex hormone testosterone has been proposed as a biological underpinning. Predictions based on current findings about the effect of testosterone on confidence levels are unclear: On the one hand, one might predict that individuals with high testosterone levels show higher levels of confidence. Body language, for example, can increase circulating testosterone levels and thereby self-confidence judgments^20^. Additionally, an increase in testosterone after losing makes people more likely to engage in a subsequent competition^21^. Furthermore, an increase in testosterone leads to a greater willingness to take financial risk^22^. Testosterone and cortisol are also reported to increase financial risk taking, which may destabilize markets^23^. Some studies also apply the digit ratio method (the ratio between the length of the index finger to the length of the ring finger, DR) to study the relation between confidence levels and testosterone. It has been reported in the literature that DR is a negatively correlated bio-marker of prenatal testosterone exposure^24^. In a recent study, for example, it has been shown that preschool children with low DR are overconfident in motor skill tasks^25^. On the other hand, individuals with higher testosterone levels may show lower levels of confidence in certain contexts. Financial traders with low DR, for example, earn higher long-term returns and stay longer in the market^26^. This may suggest that their judgments seem to be more accurate, i.e., less biased. Similarly, males with low DR are less likely to overestimate their actual performance in the “Tower of Hanoi” puzzle.

We particularly focus on the relationship between performance prediction accuracy and DR, which is one of three main methods for investigating testosterone’s effects on behavior. Many studies examine how circulating testosterone impacts decision making by either measuring its levels from saliva samples or by exogenously manipulating it^27^. Alternatively, the ratio between the lengths of the second and the fourth digits of hands has been proposed as bio-marker of prenatal testosterone exposure, which is thought to affect brain development and also sensitivity to circulating androgens^24^,^28^, and thereby more plausibly explains inter-individual differences in behavior. The relationship between DR and prenatal testosterone is negative, i.e., a low DR indicates a high level of prenatal testosterone exposure and vice versa^29^. Testosterone levels during early development can influence subsequent sex-typical behavior, such as overconfidence^30^. Furthermore, there is evidence for a negative relationship between DR and risk taking^31^,^32^, although there also exist null results in the literature^23^. We apply the DR as a bio-marker for prenatal testosterone exposure and study its relation to confidence levels.

In our study, participants were asked to answer the seven-item Cognitive Reflection Test^33^ (CRT). The seven-item CRT is an extension of the three-item CRT^34^. As dual system theories indicate, there are two cognitive systems that can be employed in human decision making. System 1 requires less effort and it is therefore fast but relies more on heuristics. System 2, on the other hand, requires more reasoning and time. System 1 reasoning yields intuitive but wrong answers on the CRT; thus employing System 2 is necessary for correct answers^35^. For example, the first item of the CRT is “A bat and a ball cost $1.10. The bat costs $1.00 more than the ball. How much does the ball cost?” Although the intuitive wrong answer is 10 cents, the correct answer is 5 cents. The full task can be found in the supplementary materials.

After completing the CRT, participants estimate their number of correct answers and the average number of correct answers among all participants in their session. This enabled us to study two aspects of overconfidence: the belief in one’s own performance relative to a) one’s actual performance and b) to one’s belief in the group’s performance. Moore and Healy refer to the former as overestimation and the latter as overplacement. We discuss the terminology in the discussion section. In half of our sample, both the task and the accuracy of guesses were incentivized, while in the other half they were not. This implies that in half of our sample wrong answers and inaccurate beliefs were “costless”, while in the other half they were not.

Our decision to explore the interaction between incentives, confidence levels, and DR is motivated by two particular concerns. Firstly, there is a folk tradition within experimental economics that questions whether phenomena from the behavioral decision research literature persist when it becomes costly to persist in them – i.e., when incentives are introduced^36^,^37^. Specific to confidence levels, relatively little overconfidence has been found when incentives for accurate guesses are given and subjects are given repeated feedback about performance^38^. Moore and Healy (2008) show that people tend to be overconfident for difficult tasks and underconfident for easy tasks^5^. Their design incentivizes guesses with a quadratic scoring rule, however, making it unclear whether this is simply due to subject risk aversion^39^. Moreover, the relation between testosterone and behavior is reported to be context-dependent^40^,^41^. Given that compelling links exist between testosterone and both confidence levels and incentives, it makes sense to explore their interaction.

## Methods

### Participants

We recruited 146 men and 139 women from the student population of the Kiel University (N = 285, mean age = 24.0 years). The experiment was organized and recruited with the software hroot^42^. Participants met in groups of 15 and were randomly assigned to seats in a classroom. At the beginning of the experiment, subjects were given general instructions about the procedure, which were followed by the experimental task described below. After the experiment, participants were invited one by one to a separate room to receive their payment and to scan both of their hands.

### Experimental Task

Participants were asked to answer the seven-item CRT within 10 minutes. After 10 minutes, the experimenters collected all answer sheets in order to prevent participants from making any changes on the task sheets. Then, they were asked to estimate their number of correct answers and the average number of correct answers within the group. It was announced that the group’s performance would be rounded to the nearest integer. In about half of our sample, each correct answer on the CRT was awarded with 0.5€ and each correct guess about one’s own and others’ scores was rewarded with 2€. This information was given in the corresponding task sheets separately to avoid strategic behavior.

### Digit Ratio

Following common recommendations^43^, both hands were scanned with a high-resolution scanner (Epson V370 Photo). To determine DR, we measured the lengths of the index and ring digits on both hands from basal crease to the fingertip using the computer software Adobe Photoshop® (Adobe Systems Inc., San Jose, USA).

Two independent raters measured the digit lengths. The DRs obtained from both raters had very high intraclass correlations (0.983 for the right hand DR and 0.977 for the left and DR). We averaged the raters’ measurements to conduct our analysis.

Although the DR studies often focus on the right hand data and a meta-analysis concludes that the right hand ratio is a better indicator, there are also numerous papers employing left hand ratios or even the means of both^44^. We study the data for both hands separately but focus on interpreting the right hand DR results. Results gathered from right and left hand DRs are consistent.

### Confidence Scores

An *overestimation score* was calculated for each individual as the difference between the individual’s estimate about her number of correct answers on the CRT and her true number of correct answers. Similarly, an *overplacement score* was calculated for each individual as the difference between the individual’s estimate about her number of correct answers on the CRT and her estimate about others’ performance.

### Ethics Statement

All participants of the experiment were informed about the content and the protocol of the study before they participated. Their anonymity was preserved by assigning them a randomly generated code that cannot be associated with any personal information or decisions. As is standard in economic experiments, no ethical concerns were involved other than preserving the anonymity of the participants. Furthermore, each participant was individually briefed about the DR measurement. This briefing included a general overview about testosterone-related studies in the social sciences and the assured anonymity of their data. We made it clear that hand scans are not related to any identifying information and that the scans would not be shared with third parties under any circumstance and will be deleted immediately after the finalization of the study. Participation in the experiment and scanning were completely voluntary. The whole protocol was in accordance with the Declaration of Helsinki and conformed to the ethical guidelines of the Kiel University Experimental Economics Lab, where it was approved by the lab manager.

## Results

### Descriptive Statistics

Subjects in all conditions are substantially overconfident in themselves on average. While actual performance on the seven-item CRT was 4.24 correct answers on average, subjects thought that they answered 5.44 questions correctly on average. This difference (i.e., overestimation) is significant according to a Wilcoxon signed-rank test (*p *= 0.000). People also estimated their own performance to be significantly better than others’, which they predicted at 5.12 correct answers. A signed-rank test also rejects equality between expected own and other performance, indicating overplacement in the overall sample (*p *= 0.000).

Incentives lead to both higher performance and higher expectations of performance. Average correct answers to the CRT are 4.04 without incentives and 4.46 with (rank-sum *p *= 0.033). Expected own performance increases from 5.18 correct answers to 5.74 correct answers (rank-sum *p *= 0.000). Note that incentives actually increase overconfidence, though not significantly (rank-sum *p *= 0.692). The expected performance of others remains unchanged at an average of 5.12 in both incentive conditions.

There are additionally gender differences in performance and confidence relative to others. The average performance of women was 3.68 questions answered correctly, while men answered 4.76 questions correctly on average. This difference is significant according to a Wilcoxon rank-sum test (*p *= 0.000). This gender effect is in line with the previous literature^34^,^45^. Men and women display similar levels of overconfidence, with women estimating themselves as answering 1.29 more questions correctly than actual performance and men overestimating by 1.10 questions. These are not significantly different (rank-sum *p *= 0.373). When we focus on the two treatments, this result remains both for incentivized and un-incentivized conditions (rank-sum *p *= 0.908 and *p *= 0.282 respectively). Men are, however, significantly more likely to say they will perform better than others (on average 0.639 questions better) than are women (who say they will perform 0.029 questions worse than others on average the difference between genders has a rank-sum *p *= 0.000). This result is valid for both incentivized and un-incentivized treatments separately as well (rank-sum *p *= 0.000 for both).

**Figure 1:**
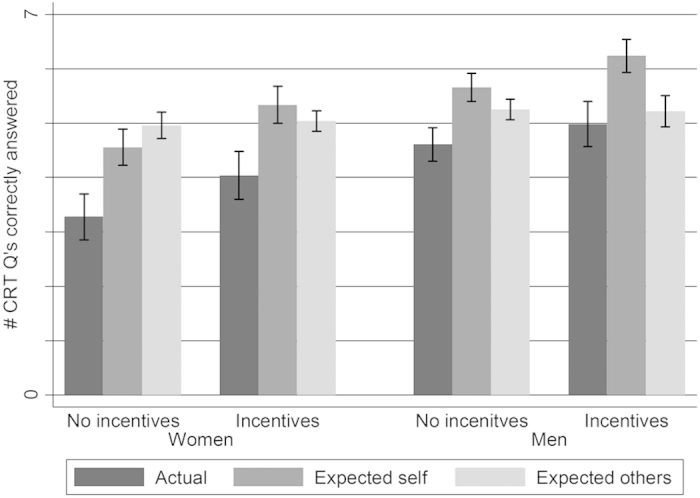
Actual and expected CRT performance by gender and incentive condition.

Women appear to respond more to incentives than do men, but this difference is not significant. Women increase their performance from 3.28 to 4.04 questions answered correctly, while men increase theirs from 4.61 to 4.98. A linear regression estimating the interaction between gender and incentive conditions has a two-tailed p-value of 0.331 on the interaction coefficient (robust standard errors are estimated). A full breakdown of results by gender and incentives treatment is shown in Figure 1.

### Digit ratios

In our sample, men had a mean right hand DR of 0.956 with a standard deviation of 0.0281, whereas women had a right hand DR of 0.967 with a standard deviation of 0.0362. This difference is significant (rank-sum *p *= 0.007). For the left hands, the mean among women is 0.970 with a standard deviation of 0.0341, and the male mean is 0.9610 with a standard deviation of 0.0281 (rank-sum *p *= 0.004). The correlation between the right and left hand DRs is 0.727 for men and 0.765 for women. Supplementary materials Figure S1 gives a fuller picture of how the distribution of right hand DR varies by gender, with women having a DR much closer to 1 but also much more variable, and men clustering more around 0.96. It should also be noted that there is no relationship between DR and CRT performance (see Supplementary Table S1).

Ultimately we are interested in prenatal testosterone exposure, for which the right hand and the left hand DRs are noisy indicators. Traditionally, the right hand DR is a more reliable indicator for testosterone exposure^44^. We therefore focus on interpreting the right hand results, though we report all regression results using the left hand DR as well. These are consistent with, though less precisely estimated than, the right hand relationships.

Table 1 estimates the relationship between DR, overestimation, and overplacement using ordinary least squares regression analysis. Because the DR variable has been standardized, the coefficients on *dr* represent the expected effect of a one-standard-deviation increase in DR on the outcome variable at the mean of the DR distribution. Stars indicate significance at the 10% level for a single star, at the 5% level for two stars, or at the 1% level for three.

Indicators and interaction terms have been added to estimate effects separately by gender and incentive condition. We do this to convey the maximum amount of information, though the resulting estimates require care to interpret. Here the excluded category is men in the no incentives treatment. Therefore the coefficient on *dr* alone represents the expected change in the outcome variable for a one standard deviation increase in DR among men in the no incentives condition. The expected change in the outcome variable for an increase of one standard deviation among women in the no incentives condition is therefore the coefficient on *dr* plus the coefficient on *female* X *dr*. Likewise for men in the incentives treatment the expected change in the outcome variable from a one standard deviation increase in DR is represented by the sum of the coefficient on *dr* plus the coefficient on *incentives* X *dr*; and finally the expected change in the outcome from a one standard deviation increase in DR among women in the incentives treatment is represented by the sum of the coefficients on *dr, female* X *dr*, and *female* X *dr* X *incentives*.

The first two columns estimate the impact of DR on overestimation, defined as the difference between expected own CRT score and actual performance, controlling for actual performance. The first finding here is that for both genders in both conditions, more poorly performing people are more overconfident (by about a half point for each full point of actual performance, on average). In some sense this is mechanical, as it is not possible to be overconfident about a perfect score, for example.

We also see that men with lower DR are significantly more overconfindent in their abilities (also by roughly a quarter point for each standard deviation in DR, *p*-value 0.001), but only in the no incentives condition. It is interesting to contrast the impact of incentives on overestimation for men at the mean of the DR distribution with the impact of incentives on men one standard deviation lower in the DR distribution. While men at the mean of the distribution become *more* (over)confident on average when incentives are introduced (by over 0.4 correct answers, *p*-value 0.015), men with low DR in the incentives condition are not significantly more confident in their abilities compared to low DR men in the no-incentives condition, (*p*-value 0.862). To see this in the regression, subtract the coefficient on *incentives* X *dr* from that on *incentives*. Incentives also reverse the expected difference between a man at the mean of the DR distribution and one who is a standard deviation below. Whereas without incentives the low-DR man was 0.287 points more confident on average (*p*-value 0.001), with incentives the low-DR man is 0.173 points *less* confident than the man at the mean of the distribution (adding the coefficients on *dr* with that on *incentives* X *dr*, though the *p*-value is only 0.215).

Note that none of these effects are seen among women. Neither a main effect of *dr* nor its interaction with the incentives treatment significantly predicts overestimation among women. Finally, while we did not see non-parametric evidence of differences in overestimation across genders, the regressions are picking up the expected finding that women are on average less confident in their own abilities than men.

The second two columns of Table 1 show a similar pattern. Here overplacement, defined as the difference between participants’ own expected performance and the expected performance of others, is regressed on DR, controlling for their guess about other’s performance. There is a strong impact of guess about others’ scores on relative confidence, similarly reflecting the fact that the higher one esteems others, the less possible it is to place oneself above them. Like with overestimation, men with higher DR are more likely to estimate their performance relative to others as higher, but only in the no incentives condition. We do not however see an interaction effect between overplacement and incentives among low-DR males (second two columns of Table 1).

**Figure 2:**
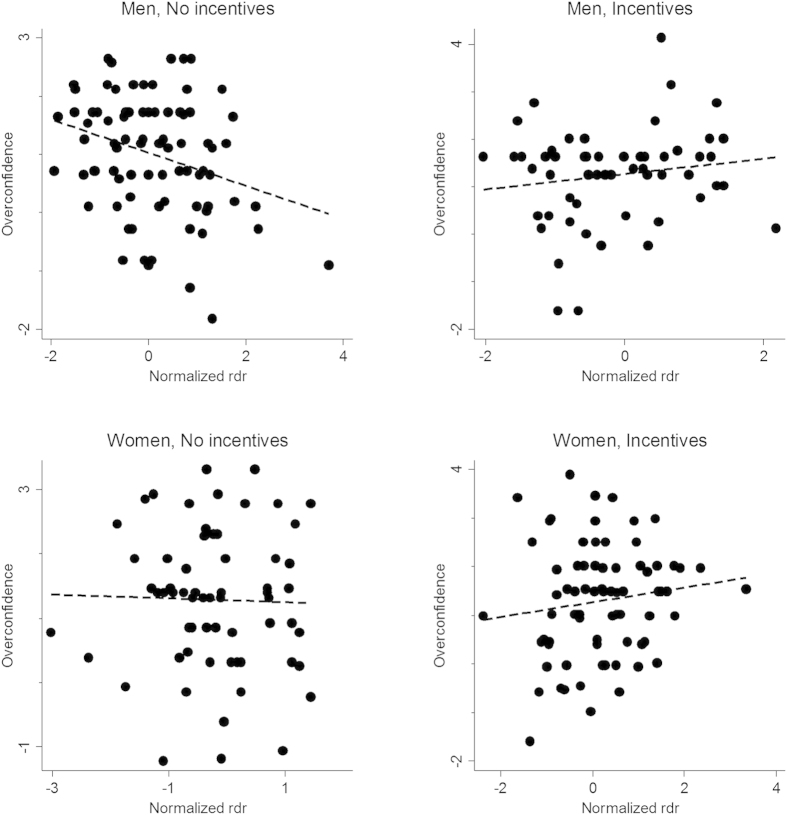
Impact of DR on overestimation across gender and incentive condition.

To visualize the main results we have plotted the regression from the second column of Table 1 (overestimation on right hand DR) as a series of bivariate graphs in Figure 2. Similar graphs for right hand DR on overplacement may be found in Supplementary Figure S2.

## Discussion

Without monetary incentives, we find that lower DR increases both average overestimation and overplacement in men. With monetary incentives, however, lower DR increases the accuracy of estimates about own performance among men on average, i.e., it reduces overconfidence. This result indicates that male participants with low DR are more sensitive towards changes in incentives and adapt their strategies accordingly. This result is discussed in the following part of the paper. The first part of the discussion sets our main results into perspective to the existing literature on the effect of testosterone on overconfidence and how it might help to explain seemingly opposing findings. The second part relates our findings to a limited literature on economic behavior and prenatal testosterone exposure. In the third part, we provide additional clarification for some of our results. Finally, potential limitations of our study are discussed.

Firstly, we outlined two competing hypotheses regarding the effect of testosterone on overconfidence in the introduction. While individuals with high levels of testosterone tend to be more overconfident in some contexts, they are less overconfident in others. Our results provide a potential explanation for these seemingly opposing findings. The main implication is that individuals with low DR are more sensitive towards changes in the incentive structure. This potentially explains why financial traders with low DR earn higher long-term returns and stay longer in the market^26^. Overconfident beliefs in financial markets may be disadvantageous, because overconfident traders trade too much and thereby reduce their net earnings^16^. In our experimental setting, men with low DR have less accurate predictions without incentives and more accurate predictions under incentives. This finding is in line with the previously outlined studies. On the other hand, body language can increase circulating testosterone levels and thereby confidence judgments^20^. We find that without monetary incentives, men with low DR overestimate their scores more. One possible explanation could be that they receive some form utility from a positive image of being better than their competitors. The positive impact of overconfidence on human well-being has already been discussed in the literature^12^,^13^. It seems that with monetary incentives, however, men with low DR value monetary profits higher than a positive self-image. This potentially explains their shift in behavior. Da Silva *et al*., by contrast, apply an un-incentivized task and find a negative correlation between DR and overconfidence^25^ in pre-school children.

Secondly, our findings shed light on the relationship between testosterone and economic behavior. Several studies in the literature show that DR correlates with risk-taking preferences^31^,^32^ and also with incentives. Yet, those working papers on incentives and DR have not been published to this date.

Thirdly, several of our results need further clarification. While subjects’ estimates about their own performance respond in significantly different ways across incentive schemes and DR, it seems puzzling why low-DR male subjects show the same increase in confidence relative to others in the incentives treatment as other subjects. Those with mean DR tend to become more confident relative to others when incentives are introduced. Fig. 1 may give some indication for why this is. We see that, on average, people perform better *and* they think they perform better when incentives are introduced, but their evaluation of others’ performance remains unchanged with the introduction of incentives. This naturally manifests as overplacement. However the low-DR males slightly lower their guesses about their own performance, and so must be lowering their estimate of others’ performance by comparatively more – since they display the same increase in overplacement as the other subjects.

We can also show that the interaction between DR and incentives is not due solely to differential changes in performance across incentive schemes. Supplementary Table S1 estimates the effect of DR on absolute CRT performance. There is no significant effect of DR on performance in either treatment, nor is there a significant difference between treatments. In contrast, Bosch-Domench *et al*.^46^ show a negative relationship between DR and performance in the 3 item-CRT in a Spanish sample^46^.

It is also interesting to note that DR has no significant impact on overestimation or overplacement among female subjects. We might expect that similar hormonal mechanisms are at work for both genders, but perhaps the relationship between DR and overconfidence among males’ results from an interaction between prenatal testosterone exposure and differences in socialization across genders.

Our results may also be related to the connection between prenatal and adult testosterone. Current knowledge on prenatal testosterone points out organizational effects on endocrine system (among others) and these effects may be seen in adults. One possible reason for our findings may be the short term spikes in circulating testosterone in competitive situations. In particular DR may be a negative marker for the amplitude of these peaks and sensitivity to testosterone itself. These peaks are much more likely to be found in males than females^41^. Crewther *et al*.^47^ also discuss the links between DR and sex-dependent challenge-induced peaks in testosterone^47^. The incentive condition may represent a challenge which results in an increase in testosterone in men but not women. This then may explain the difference between the DR-overconfidence correlation in the non-incentive condition (when testosterone is at background levels) and the DR-overconfidence correlation in the incentive condition (when testosterone may be elevated in a short-term peak).

We tried to use the terminology in a careful manner in the paper. The majority of the studies in the literature use the term, *overconfidence*. Yet, it should be noted that, although most of the participants are overconfident about their own performance, there also exist under-confident participants as well as those who estimated their correct answers correctly. 10.67% of the sample is under-confident and 18% made correct guesses in the un-incentivized condition. In the incentivized condition, under-confident participants are 6.02% of the sample and 23.30% estimated their scores correctly. For this reason, we use the word *confidence* as a relatively neutral term where necessary. For the dependent variable names on the other hand, we prefer to use the standard definition of Moore and Healy^5^. They differentiate between overly optimistic beliefs (1) about one’s performance relative to the actual performance (*overestimation*), (2) about one’s performance relative to others (*overplacement*), and (3) about the accuracy of one’s private information (*overprecision*).

Finally, limitations of our study should be discussed. Our participant sample consists solely of university students and therefore suffers from the well-known representativeness bias of experimental studies^48^. Furthermore, studies on DR have several common drawbacks. DR is sensitive to ethnic differences^49^. Our sample however contains only European Caucasian participants. It is a usual protocol to focus on the major subsample of the study or divide the large, multi-ethnic samples in smaller groups^50^. Another issue for DR studies is the repeatability of the measurements. Since the measurements of the images are done manually by human evaluators it is possible that measurements of different evaluators may not highly correlate in some datasets. One crucial precaution for this issue is the scanning quality. Following a standard scanning protocol as well as using a high resolution scanner is helpful. We repeat measurement of the digits, another recommended procedure. Additionally, automatized measurement methods and algorithms are being developed^51^. It is also worth mentioning that our results cannot be taken as conclusive for the biological underpinnings of overconfidence and the effects of incentives. According to Dual Inheritance Theory, human behavior is influenced by both social environment and genes. DR is shown to have genetic underpinnings^52^. Although investigating these underpinnings of human behavior is important, the environmental factors shaping human behavior cannot be a neglected.

## Additional Information

**How to cite this article**: Neyse, L. *et al*. Overconfidence, Incentives and Digit Ratio. *Sci. Rep.* **6**, 23294; doi: 10.1038/srep23294 (2016).

## Supplementary Material

Supplementary Information

## Figures and Tables

**Table 1:** Impact of DR’s interaction with incentives on estimation.

Dep var	Overestimation		Overplacement	
	Left hand	Right hand	Left hand	Right hand
*crt_correct*	−0.553***	−0.557***	–	–
	(0.0417)	(0.0425)	–	–
*guess_other*	–	–	−0.344***	−0.351***
	–	–	(0.0871)	(0.0868)
*dr*	−0.134	−0.287***	−0.170	−0.274**
	(0.106)	(0.0894)	(0.120)	(0.116)
*female*	−0.499***	−0.517***	−0.900***	−0.914***
	(0.178)	(0.181)	(0.200)	(0.203)
*incentives*	0.453***	0.420**	0.606***	0.573***
	(0.168)	(0.171)	(0.175)	(0.175)
*female*	0.0582	0.255	0.109	0.239
X *dr*	(0.163)	(0.169)	(0.206)	(0.202)
*incentives*	0.478**	0.460***	0.182	0.201
X *dr*	(0.215)	(0.166)	(0.175)	(0.177)
*female*	−0.0157	−0.00880	0.106	0.102
X *incentives*	(0.255)	(0.263)	(0.270)	(0.275)
*female* X *dr*	−0.302	−0.263	0.107	0.110
X *incentives*	(0.293)	(0.250)	(0.292)	(0.275)
constant	3.58	3.62	2.20	2.25
	(0.224)	(0.226)	(0.488)	(0.482)
N	281	281	280	280
*R*^2^	0.440	0.447	0.210	0.220

Note: Ordinary least squares regression analysis: Robust standard errors are indicated in brackets. Independent variables are *crt_correct* for the number of correct answers in the CRT, *dr* for standardised DR, *female* is a dummy variable with 1 for women, incentives is a dummy variable with 1 for the incentive condition. We had 285 observations. This number is reduced here due to missing information for some participants.

## References

[b1] Festinger L. A Theory of Social Comparison Processes. Hum. Relations 7, 117–140 (1954).

[b2] Niederle M. & Vesterlund L. Do Women Shy Away From Competition? Do Men Compete Too Much? Q. J. Econ. 122, 1067–1101 (2007).

[b3] Garcia S. M., Tor, a. & Schiff, T. M. The Psychology of Competition: A Social Comparison Perspective. Perspect. Psychol. Sci. 8, 634–650 (2013). 26173228 10.1177/1745691613504114

[b4] Mannes A. & Moore D. I know i’m right! A behavioural view of overconfidence. Significance 10, 10–14 (2013).

[b5] Moore D. a & Healy, P. J. The trouble with overconfidence. Psychol. Rev. 115, 502–517 (2008). 18426301 10.1037/0033-295X.115.2.502

[b6] Wrangham R. Is Military Incompetence Adaptive? Evol. Hum. Behav. 20, 3–17 (1999).

[b7] Bénabou R. & Tirole J. Self-confidence and personal motivation. Q. J. Econ. 871–915 (2002).

[b8] McKay R. T. & Dennett D. C. The evolution of misbelief. Behav. Brain Sci. 32, 493–510; discussion 510–561 (2009). 20105353 10.1017/S0140525X09990975

[b9] Johnson D. D. P. & Fowler J. H. The evolution of overconfidence. Nature 477, 317–320 (2011). 21921915 10.1038/nature10384

[b10] Stankov L., Lee J., Luo W. & Hogan D. J. Confidence: A better predictor of academic achievement than self-efficacy, self-concept and anxiety? Learn. Individ. Differ. 22, 747–758 (2012).

[b11] Johnson D. D. P., Weidmann N. B. & Cederman L.-E. Fortune favours the bold: an agent-based model reveals adaptive advantages of overconfidence in war. PLoS One 6, e20851 (2011). 21731627 10.1371/journal.pone.0020851 PMC3123293

[b12] Taylor S. E. & Brown J. D. Illusion and well-being: a social psychological perspective on mental health. Psychol. Bull. 103, 193–210 (1988). 3283814

[b13] Taylor S. E. & Brown J. D. Positive illusions and well-being revisited: separating fact from fiction. Psychol. Bull. 116, 21–27; discussion 28 (1994). 8078971 10.1037/0033-2909.116.1.21

[b14] Plous S. The psychology of judgement and decision making. (Mcgraw-Hilll, 1993).

[b15] Della Vigna S. & Malmendier U. Paying Not to Go to the Gym. Am. Econ. Rev. 96, 694–719 (2006).

[b16] Barber B. M. & Odean T. Boys will be boys: Gender, overconfidence, and common stock investment. Q. J. Econ. 261–292 (2001).

[b17] Camerer C. & Lovallo D. Overconfidence and excess entry: An experimental approach. Am. Econ. Rev. 89, 306–318 (1999).

[b18] De Bondt W. F. M. & Thaler R. H. Chapter 13 Financial decision-making in markets and firms: A behavioral perspective. Handbooks Oper. Res. Manag. Sci. 9, 385–410 (1995).

[b19] Lundeberg M. A., Fox P. W. & Punc-cohar-J. Highly confident but wrong: Gender differences and similarities in confidence judgments. J. Educ. Psychol. 86, 114–121 (1994).

[b20] Carney D. R., Cuddy A. J. C. & Yap A. J. Power posing: brief nonverbal displays affect neuroendocrine levels and risk tolerance. Psychol. Sci. a J. Am. Psychol. Soc. / APS 21, 1363–1368 (2010). 10.1177/0956797610383437 20855902

[b21] Mehta P. H. & Josephs R. a. Testosterone change after losing predicts the decision to compete again. Horm. Behav. 50, 684–692 (2006). 16928375 10.1016/j.yhbeh.2006.07.001

[b22] Apicella C. L., Dreber A. & Mollerstrom J. Salivary testosterone change following monetary wins and losses predicts future financial risk-taking. Psychoneuroendocrinology 39, 58–64 (2014). 24275004 10.1016/j.psyneuen.2013.09.025

[b23] Cueva C. . Cortisol and testosterone increase financial risk taking and may destabilize markets. Sci. Rep. 5, 11206 (2015). 26135946 10.1038/srep11206 PMC4489095

[b24] Manning J. T., Scutt D., Wilson J. & Lewis-Jones D. I. The ratio of 2nd to 4th digit length: a predictor of sperm numbers and concentrations of testosterone, luteinizing hormone and oestrogen. Hum. Reprod. 13, 3000–3004 (1998). 9853845 10.1093/humrep/13.11.3000

[b25] Da Silva S., Moreira B. & Da Costa N. Jr Handedness and digit ratio predict overconfidence in cognitive and motor skill tasks in a sample of preschoolers. Econ. Bull. 35, 1087–1097 (2015).

[b26] Coates J. M., Gurnell M. & Rustichini A. Second-to-fourth digit ratio predicts success among high-frequency financial traders. Proc. Natl. Acad. Sci. USA. 106, 623–628 (2009). 19139402 10.1073/pnas.0810907106 PMC2626753

[b27] Apicella C. L. . Testosterone and financial risk preferences. Evol. Hum. Behav. 29, 384–390 (2008).

[b28] Goy R. & McEwen B. Sexual Differentiation of the Brain. Cambridge. (MIT Press).

[b29] Lutchmaya S., Baron-Cohen S., Raggatt P., Knickmeyer R. & Manning J. T. 2Nd To 4Th Digit Ratios, Fetal Testosterone and Estradiol. Early Hum. Dev. 77, 23–28 (2004). 15113628 10.1016/j.earlhumdev.2003.12.002

[b30] Constantinescu M. & Hines M. Relating Prenatal Testosterone Exposure to Postnatal Behavior in Typically Developing Children: Methods and Findings. Child Dev. Perspect. 6, 407–413 (2012).

[b31] Brañas-Garza P. & Rustichini A. Organizing effects of testosterone and economic behavior: Not just risk taking. PLoS One 6, 1–17 (2011). 10.1371/journal.pone.0029842 PMC3248440 22242144

[b32] Garbarino E., Slonim R. & Sydnor J. Digit ratios (2D:4D) as predictors of risky decision making for both sexes. J. Risk Uncertain. 42, 1–26 (2011).

[b33] Toplak M. E., West R. F. & Stanovich K. E. Assessing miserly information processing: An expansion of the Cognitive Reflection Test. Think. Reason. 20, 147–168 (2014).

[b34] Frederick S. Cognitive Reflection and Decision Making. J. Econ. Perspect. 19, 25–42 (2005).

[b35] Kahneman D. & Frederick S. In Heuristics and Biases (eds. Gilovich T., Griffin D. & Kahneman D.) 49–81 (Cambridge University Press, 2002).

[b36] Grether D. M. & Plott C. R. Economic Theory of Choice and the Preference Reversal Phenomenon. Am. Econ. Rev. 69, 623–638 (1979).

[b37] Myagkov M. & Plott C. R. Exchange Economies and Loss Exposure: Experiments Exploring Prospect Theory and Competitive Equilibria in Market Environments. Am. Econ. Rev. 87, 801–828 (1997).

[b38] Clark J. & Friesen L. Overconfidence in forecasts of own performance: An experimental study. Econ. J. 119, 229–251 (2009).

[b39] Winkler R. L. & Murphy A. H. Nonlinear Utility and the Probability Score. J. Appl. Meteorol. 9, 143–148 (1970).

[b40] Millet K. & Dewitte S. The presence of aggression cues inverts the relation between digit ratio (2D:4D) and prosocial behaviour in a dictator game. Br. J. Psychol. 100, 151–162 (2009). 18590604 10.1348/000712608X324359

[b41] Manning J., Kilduff L., Cook C., Crewther B. & Fink B. Digit Ratio (2D:4D): A Biomarker for Prenatal Sex Steroids and Adult Sex Steroids in Challenge Situations. Front. Endocrinol. *(Lausanne)*. 5, 9 (2014). 10.3389/fendo.2014.00009 PMC3906590 24523714

[b42] Bock O., Baetge I. & Nicklisch A. hroot: Hamburg Registration and Organization Online Tool. Eur. Econ. Rev. 71, 117–120 (2014).

[b43] Kemper C. J. & Schwerdtfeger A. Comparing indirect methods of digit ratio (2D:4D) measurement. Am. J. Hum. Biol. 21, 188–191 (2009). 18988284 10.1002/ajhb.20843

[b44] Hönekopp J. & Watson S. Meta-analysis of digit ratio 2D:4D shows greater sex difference in the right hand. Am. J. Hum. Biol. 22, 619–630 (2010). 20737609 10.1002/ajhb.21054

[b45] Oechssler J., Roider A. & Schmitz P. W. Cognitive abilities and behavioral biases. J. Econ. Behav. Organ. 72, 147–152 (2009).

[b46] Bosch-Domènech A., Brañas-Garza P. & Espín A. M. Can exposure to prenatal sex hormones (2D:4D) predict cognitive reflection? Psychoneuroendocrinology 43, 1–10 (2014). 24703165 10.1016/j.psyneuen.2014.01.023

[b47] Crewther B., Cook C., Kilduff L. & Manning J. Digit ratio (2D:4D) and salivary testosterone, oestradiol and cortisol levels under challenge: Evidence for prenatal effects on adult endocrine responses. Early Hum. Dev. 91, 451–456 (2015). 26025335 10.1016/j.earlhumdev.2015.04.011

[b48] Levitt S. D. & List J. A. What Do Laboratory Experiments Measuring Social Preferences Reveal about the Real World? J. Econ. Perspect. 21, 153–174 (2007).

[b49] Manning J. T., Stewart a., Bundred P. E. & Trivers R. L. Sex and ethnic differences in 2nd to 4th digit ratio of children. Early Hum. Dev. 80, 161–168 (2004). 15500996 10.1016/j.earlhumdev.2004.06.004

[b50] Galizzi M. M. & Nieboer J. Digit ratio (2D:4D) and altruism: evidence from a large, multi-ethnic sample. Front. Behav. Neurosci. 9, 41 (2015). 25755639 10.3389/fnbeh.2015.00041 PMC4337370

[b51] Sandnes F. E. An Automatic Two-hand 2D:4D Finger-ratio Measurement Algorithm for Flatbed Scanned Images. in *International Conference on Systems, Man, and Cybernetics* 1203–1208 (IEEE, Hong Kong, 2015). doi: 10.1109/SMC.2015.215

[b52] Paul S. N., Kato B. S., Cherkas L. F., Andrew T. & Spector T. D. Heritability of the second to fourth digit ratio (2d:4d): A twin study. Twin Res. Hum. Genet. 9, 215–219 (2006). 16611491 10.1375/183242706776382491

