# Supplementary material for: Overconfidence, Incentives and Digit Ratio
Source: Sci Rep. 2016 Apr 4;6:23294. doi: 10.1038/srep23294 (PMC4819182; doi:10.1038/srep23294)
Supplement: Supplementary Information [file srep23294-s1.doc]

**Overconfidence, Incentives and Digit ratio**

Levent Neyse, Steven Bosworth, Patrick Ring and Ulrich Schmidt

**(Supplementary Materials)**

**Table of Contents**

Supplementary 1: Cognitive Reflection Test (Toplak et al., 2014)

Figure S1: Distribution of digit ratios by gender

Figure S2: Impact of DR on relative placement across gender and incentive condition

Table S1: Impact of digit ratio on CRT questions answered correctly

**Supplementary 1**

**Cognitive Reflection Test (Toplak et al., 2014)**

(1) A bat and a ball cost $1.10 in total. The bat costs a dollar more than the ball. How much does the ball cost? ____ cents [Correct answer = 5 cents; intuitive answer = 10 cents]

(2) If it takes 5 machines 5 minutes to make 5 widgets, how long would it take 100 machines to make 100 widgets? ____ minutes [Correct answer = 5 minutes; intuitive answer = 100 minutes]

(3) In a lake, there is a patch of lily pads. Every day, the patch doubles in size. If it takes 48 days for the patch to cover the entire lake, how long would it take for the patch to cover half of the lake? ____ days [Correct answer = 47 days; intuitive answer = 24 days]

(4) If John can drink one barrel of water in 6 days, and Mary can drink one barrel of water in 12 days, how long would it take them to drink one barrel of water together? _____ days [correct answer = 4 days; intuitive answer = 9]

(5) Jerry received both the 15th highest and the 15th lowest mark in the class. How many students are in the class? ______ students [correct answer = 29 students; intuitive answer = 30]

(6) A man buys a pig for $60, sells it for $70, buys it back for $80, and sells it finally for $90. How much has he made? _____ dollars [correct answer = $20; intuitive answer = $10]

(7) Simon decided to invest $8,000 in the stock market one day early in 2008. Six months after he invested, on July 17, the stocks he had purchased were down 50%. Fortunately for Simon, from July 17 to October 17, the stocks he had purchased went up 75%. At this point, Simon has: a. broken even in the stock market, b. is ahead of where he began, c. has lost money [correct answer = c, because the value at this point is $7,000; intuitive response = b].

**Supplementary Figure S1** **Distribution of digit ratios by gender**

Figure S1: Distribution of digit ratios by gender

**Supplementary Figure S2** **Impact of DR on relative placement across gender and incentive condition**

Figure S2: Impact of DR on overplacement across gender and incentive condition

**Supplementary Table S1: Impact of digit ratio on CRT questions answered correctly**

| Dep var: | CRT Performance | |
| --- | --- | --- |
|  | Left hand | Right hand |
| *dr* | -.145 | -.146 |
|  | (.148) | (.150) |
| *female* | -1.35*** | -1.37*** |
|  | (.268) | (.281) |
| *incentives* | .398 | .367** |
|  | (.270) | (.267) |
| *female* | .0590 | .0109 |
| X *dr* | (.291) | (.316) |
| *incentives* | .155 | -.0300 |
| X *dr* | (.309) | (.269) |
| *female* | .334 | .347 |
| X *incentives* | (.409) | (.419) |
| *female* X *dr* | .404 | .569 |
| X *incentives* | (.440) | (.433) |
| constant | 4.62 | 4.62 |
|  | (.159) | (.159) |
| N | 283 | 283 |
| *R*^2^ | .143 | .142 |

Note: Ordinary least squares regression analysis: Robust standard errors are indicated in brackets. Independent variables are *dr* for DR, *female* is a dummy variable with 1 for women, *incentives* is a dummy variable with 1 for the incentive condition. We had 285 observations. This number is reduced here due to missing information for some participants.

Table S1: Impact of digit ratio on CRT questions answered correctly
